# Therapeutic Potential of Kelp Fucoidan in Rebiosis of Gut Microflora and Immune Homeostasis in Cyclophosphamide-Induced Immunosuppressed Mice

**DOI:** 10.3390/foods14152662

**Published:** 2025-07-29

**Authors:** Yaqing Liu, Ruining Kang, Yanfei Zhao, Heng Zhang, Qingfeng Rong, Shaoxuan Yu, Yaoguang Chang, Zhengpeng Wei, Lanlan Zhu

**Affiliations:** 1 College of Agricultural Engineering and Food Science, Shandong University of Technology, Zibo 255000, China; liuyaqing510@163.com (Y.L.); kangrn0208@163.com (R.K.); 19710116454@163.com (Y.Z.); 13954072117@163.com (H.Z.); yusx89sdut@126.com (S.Y.); 2 Zichuan District Inspection and Test Center, Zibo 255100, China; 15615335002@163.com; 3 College of Food Science and Engineering, Ocean University of China, Qingdao 266000, China; changyg@ouc.edu.cn; 4 Rongcheng Taixiang Food Co., Ltd., Rongcheng 264333, China; wei_zhp@126.com

**Keywords:** kelp fucoidan, intestinal flora, short chain fatty acid, immunosuppressed mice

## Abstract

Recent studies indicate that fucoidan may play a crucial role in the metabolism and biological function of the intestinal flora. This study investigates the therapeutic potential of kelp fucoidan on the gut microbiota and immune homeostasis of cyclophosphamide-induced immunosuppressed mice. An immunosuppressive mouse model was established using cyclophosphamide, followed by administration of various kelp fucoidan doses (low-dose fucoidan: 50 mg/(kg·bw)/d, medium-dose fucoidan: 100 mg/(kg·bw)/d, and high-dose fucoidan: 150 mg/(kg·bw)/d) to the experimental groups. Changes in the gut microbiota structure were analyzed using 16S rRNA high-throughput sequencing, alongside simultaneous measurement of serum immune indicators and levels of short-chain fatty acids (SCFAs). Results indicate that kelp fucoidan significantly improved the thymus and spleen indices in immunosuppressed mice (*p* < 0.05) and elevated serum levels of IgM, IgG and IL-4. Post-kelp fucoidan intervention, there was significant alteration in microbiota ecosystem restructuring, such as proliferation in probiotics, including *Lactobacillus* and *Bifidobacterium*, while opportunistic pathogens, such as *Enterococcus* and *Escherichia coli*, decreased. Furthermore, the levels of acetic, propionic, and butyric acids in the colonic contents of the kelp fucoidan group significantly improved (*p* < 0.01). This research demonstrates that kelp fucoidan enhances immune function in immunosuppressed mice by modulating gut microbiota balance and promoting short-chain fatty acid production.

## 1. Introduction

The human intestine is a complex ecosystem with a microbiota composed of over 1000 types of microorganisms, which are particularly important for maintaining the physiological functions of the host [1]. Studies have shown that the gut microbiota plays a significant role in the pathogenesis of various diseases, including obesity, diabetes, inflammatory bowel disease, and neurodegenerative diseases, through mechanisms such as metabolic regulation and immune regulation. It is worth noting that as a controllable factor, changes in dietary structure can directly affect the composition and function of the intestinal flora, thereby regulating the host’s metabolic state and disease susceptibility [2].

Cyclophosphamide (CTX) is a potent alkylating agent that is often used as an immunosuppressant in autoimmune diseases and cancer therapies [3,4]. Studies have demonstrated that CTX causes DNA damage in macrophages and lymphocytes, thereby impairing both humoral and cellular immunity [5,6]. Furthermore, prolonged high-dose CTX administration (80–100 mg/kg, body weight) disrupts microbiota matrix and barrier integrity [7,8]. Therefore, it is of great significance to search for natural active substances that can simultaneously regulate the intestinal flora and immune function.

Recent studies have reported the potential of polysaccharides as multifunctional immunomodulatory safe compounds with minimal side effects [9,10]. These natural compounds exhibit significant therapeutic potential, especially for improving host immunity while maintaining intestinal barrier integrity [11,12]. For instance, researchers revealed effective modulation properties of yam polysaccharides toward maintaining gut microbiota in CTX-induced mice [9]. Others highlighted the potential of mulberry leaf polysaccharides in restoring microbial balance and improving intestinal overall health [13]. Likewise, polysaccharides extracted from *Millettia speciosa* exhibited ameliorating efficacy in an intestinally injured and immunosuppressed CTX-induced animal model [14].

Fucoidan is a sulfated polysaccharide derived from the ocean, featuring unique prebiotic and immunomodulatory properties [15,16,17,18]. Its unique sulfated structure, as a prebiotic, can be fermented by specific intestinal microorganisms. By regulating the proliferation of beneficial bacteria, such as Lactobacillus and Bacteroides (such as Myxophilus, Cyanobacteria and Propionibacterium) and inhibiting pathogenic bacteria (such as Gastrococcus), it maintains the balance of intestinal flora [19]. It simultaneously drives the production of short-chain fatty acids (such as butyric acid), thereby enhancing intestinal barrier function, regulating immune homeostasis, and improving host energy metabolism and inflammatory response by regulating the activity of metabolic enzymes in the microbiota [20]. While improving metabolic syndrome and intestinal malnutrition, it ultimately plays a core regulatory role in the “microbiota–host” interaction [21]. Its natural source safety and proven health benefits make it an ideal intervention substance for exploring the mechanism of the “microbiota–immune axis” [22]. A group of scientists, Cheng et al. [23], revealed the physicochemical properties of sargassum fucoidan and its effects on streptozotocin-induced diabetic mice and their gut microbiota. The study showed that fucoidan considerably modifies the microbial composition, which eventually revealed positive effects in an induced diabetic mice model. Similarly, Fang et al. [24] found that Dendrobium officinale leaf polysaccharides affected glycolipid metabolism and gut microbiota imbalance in type 2 diabetic mice. Moreover, they observed an increase in short-chain fatty acid (SCFAs) level in the colon, along with propagation of beneficial bacteria, such as *Lactobacillus* and *Bifidobacterium*, instead of reducing the proportions of *Bacteroidota* and *Firmicutes*. Sun T et al. [25] defined the positive effect of fucoidan in producing SCFAs and anti-PD-1 immunotherapy through regulating gut microbiota.

Although these findings cumulatively indicate the therapeutic potential of fucoidan, there are still key research gaps in the study of the immunomodulatory therapeutic functions of marine biological polysaccharides and their mechanisms of action on the intestinal microbiota, as well as in improving intestinal immune function and microbiota composition. Hence, the present research extracted fucoidan from kelp, and the 16S rRNA technique was applied to evaluate the diversity and composition differences of intestinal flora in the feces and colonic contents of immunosuppressed mice. It turns out that kelp fucoidan can heighten immune function in these mice by regulating intestinal flora balance and promoting the production of SCFAs.

## 2. Materials and Methods

### 2.1. Materials and Reagents

#### 2.1.1. Experimental Materials

Dried Long Qing (Central Region) kelp in April 2023, origin: Rongcheng City, Shandong Province; kelp fucoidan; 5-week-old SPF-grade BALB/C male mice (Jinan Pengyue Experimental Animal Breeding Co., LTD., Jinan, China), production license number: SCXK (Lu) 20220006; feed for mice, ordinary grade corn cob bedding (Keao Xieli Feed Co., LTD., Beijing, China).

#### 2.1.2. Main Reagents

TruSeq^®^ DNA without PCR sample (Illumina Company, San Diego, CA, USA); phosphoric acid (Sinopharm Group Chemical Reagent Co., LTD., Shanghai, China); ether (Sinopharm Group Chemical Reagent Co., LTD., Shanghai, China); acetic acid (Dr. Ehrenstorfer, North Rhine-Westphalia, Germany); propionic acid (Fluka, St. Louis, MO, USA); isobutyric acid (TMSTANDARD, Beijing, China); N-butyric acid (TMSTANDARD, Beijing, China); isovaleric acid (CNW, Shanghai, China); N-valeric acid (CNW, Shanghai, China). All other chemical reagents used were of analytical grade.

#### 2.1.3. Main Instruments

Electronic balance (FA-1004, Shanghai Sunny Hengpingke, Shanghai, China); centrifuge (TGL-16M, Xiangyi, Changsha, China); gas chromatograph mass spectrometer (Trace1310 Isq7000, Thermo, Zhengzhou, China); Illumina NovaSeq 6000 (NovaSeq 6000, Illumina, San Diego, CA, USA); high-performance liquid chromatography (UltiMate3000, Thermo, Zhengzhou, China); differential detector (OPTILAB T-rex, Wyatt, Shanghai, China); laser light scattering detector (DAWNhELEOS-Ⅱ, Wyatt, Shanghai, China).

### 2.2. Experimental Methods

#### 2.2.1. Preparation of Kelp Fucoidan

First, 10 g of dried kelp powder was soaked overnight in 100 mL of 95% (*v*/*v*) ethanol. The defatted powder was then subjected to enzymatic hydrolysis using a binary enzyme system consisting of cellulose (2.0%, *w*/*v*) and papain (2%, *w*/*v*) in distilled water at a solid to liquid ratio of 1:30 (*w*/*v*). The reaction mixture was adjusted to pH 6.0 and incubated at 40 °C for 3 h with continuous agitation to facilitate polysaccharide release. Enzymatic activity was stopped through heat inactivation at 100 °C for 10 min, followed by filtration through 200-mesh cloth to remove insoluble residues. To remove alginates, the clarified supernatant was treated with CaCl_2_ (4%, *w*/*v*) at a 4:1 ratio (*v*/*v*), and the resulting precipitates were removed through centrifugation at 3500× *g* for 15 min. The supernatant was concentrated under reduced pressure, and fucoidan was precipitated with ethanol up to a concentration of 75% (*v*/*v*), followed by incubation overnight at 4 °C. The precipitates were recovered through centrifugation at 3000× *g* for 10 min, washed sequentially with anhydrous ethanol to remove any possible residual impurities, and subsequently lyophilized to obtain purified fucoidan [26].

#### 2.2.2. Structural Characterization of Kelp Fucoidan

An appropriate amount of dry kelp fucoidan sample (about 1–2 mg) was mixed with KBr at a ratio of 1:100 (*w*/*w*), ground thoroughly and evenly, and then pressed into a transparent thin sheet. Scanning was conducted using FTIR within the wavelength range of 4000–400 cm^−1^. The main functional group structure was identified by analyzing the characteristic absorption peaks [27].

The molecular weight distribution and molecular size of kelp fucoidan were accurately determined by HPGPC-MALLS technology [28]. The sample was dissolved in a 0.1 mol/L NaNO_3_ aqueous solution containing 0.02%NaN_3_ at a final concentration of 1 mg/mL. After filtration, the sample was injected. The chromatographic columns selected were OhpakSB-805HQ and OhpakSB-803HQ. The column temperature was 45 °C, the injection volume was 100 μL, the mobile phase was A (0.1 mol/L NaNO_3_ containing 0.02%NaN_3_), the flow rate was 0.6 mL/min, and isocratic elution was performed for 75 min. The elution curve was monitored by RI, and the absolute molecular weight was determined by MALLS. Mn, Mw, D, and Rg were calculated with the aid of ASTRA 6.1 software to characterize the molecular weight distribution and conformational characteristics.

The composition of monosaccharides was analyzed by the pre-column derivatization method. First, derivatization with PMP was performed: kelp fucosyllan was hydrolyzed at 110 °C in trifluoroacetic acid for 4 h to release monosaccharides, and then reacted with PMP under alkaline conditions for 30 min. Analysis was performed using Agilent 1260 high-performance liquid chromatography under the following conditions: Dionex CarboPac PA20 chromatographic column, 30 °C, 5 μL injection volume, ammonium acetate buffer, and acetonitrile mixed mobile phase, and flow rate of 1.0 mL/min. The diode array detector detected at a wavelength of 250 nm to ensure accuracy. This method can precisely analyze the monosaccharide composition of fucoidan in kelp, supporting the research on its structure and function [28].

#### 2.2.3. Establishment and Administration of Immunocompromised Mouse Models

After one week of adaptive rearing, 25 BALB/C mice were randomly assigned into five groups based on body weight. As shown in Table 1, with five mice per group: blank control (CON), model group (CTX), low-dose fucoidan (FL), medium-dose (FM), and high-dose (FH). All mice were injected a subcutaneous injection of 50 mg/(kg·bw) cyclophosphamide once daily for five consecutive days to set up an immunosuppression model [29,30,31]. Thirty minutes post-first injection, gavage administration commenced, with the CTX group receiving normal saline (0.1 mL/10 g/d) and the FL (50 mg/(kg·bw)/d), FM (100 mg/(kg·bw)/d), and FH (150 mg/(kg·bw)/d) groups receiving their respective doses of fucoidan daily. All experimental groups were administered continuously for 14 days. After the last administration, blood was collected from the eyeballs, and the spleens and thymuses were removed for weighing and calculation of organ indices. Colonic contents were stored at −80 °C for 16S rRNA analysis. At the same time, the intestinal feces and colonic contents were collected for SCFA level determination.

#### 2.2.4. DAI Index of Immunosuppressed Mice

According to the DAI index [32] (disease activity index) of mice noted during the experimental period, the DAI evaluation criteria are shown in Table 2.

#### 2.2.5. Determination of Immune Organ Indices in Immunosuppressed Mice

The spleen and thymus were weighed.

Viscera coefficient = (viscera weight/body weight in mice) × 100(1)

#### 2.2.6. Determination of Serum Immune Indicators in Immunosuppressed Mice

The determination of IFN-γ, TNF-α, IL-6, IL-1β, IL-4, IgM, and IgG in serum was carried out on the basis of the ELISA test kit (Jiangsu Enzyme Immunoassay Industry Co., LTD, Yancheng, China, 2024012382M, 2024012332M, 2024012363M, 2024012363M, 2024012365M, 2024012358M, and 2024012357M).

#### 2.2.7. Determination of 16S rRNA in Immunocompromised Mice

Colon contents and fecal samples were collected and sent to the Stand Testing Company (Qingdao, China) for testing.

Primer information: F: ACTCCTACGGGAGGCAGCA;

R: GGACTACHVGGGTWTCTAAT.

#### 2.2.8. Determination of SCFAs in Feces and Colonic Contents of Immunosuppressed Mice

The sample pretreatment was carried out in accordance with Tan Li’s [33] method, and the treated samples were analyzed by gas chromatography–mass spectrometry. A DB-WAX column (30 m × 0.32 mm × 0.25 μm) was used at an initial temperature 60 °C, held for 3 min, then increased to 140 °C at 10 °C/min until the column temperature was controlled at 230 °C. Meanwhile, the injection port temperature ran to 250 °C, the velocity of flow in carrier gas was 1.0 mL/min, and the split ratio was 20:1. For the mass spectrometry conditions, adapting a single-ion electron bombardment (EI) source scanning mode, the ion source temperature was adjusted to 250 °C while the transmission line temperature was controlled at 230 °C, and the voltage was 70 eV. The quantitative ions were 60 and 73. The SCFA contents were calculated by the following formula:

(2) $$W=\frac{(C-C_{0})\times V\times N}{m}$$

where W is the SCFA content of a sample (mg/kg); C is the SCFA concentration in the sample testing solution (mg/L); C_0_ is the target substances’ concentration in the CON (mg/L); V is the constant volume (mL); N is the dilution factor; m is the sample quality (g).

#### 2.2.9. Data Statistical Processing

All experiments were performed in triplicate, with gut microbiota data analyzed using the Stand Company platform. SPSS 26 software was used for data processing and statistical analysis, with conspicuousness defined at *p* < 0.05 and extreme conspicuousness at *p* < 0.01. Origin 2021 was used to create diagrams, where different letters stand for prominent differences between data sets (*p* < 0.05).

## 3. Results and Discussion

### 3.1. Structural Characterization of Kelp Fucoidan

**Figure 1:**
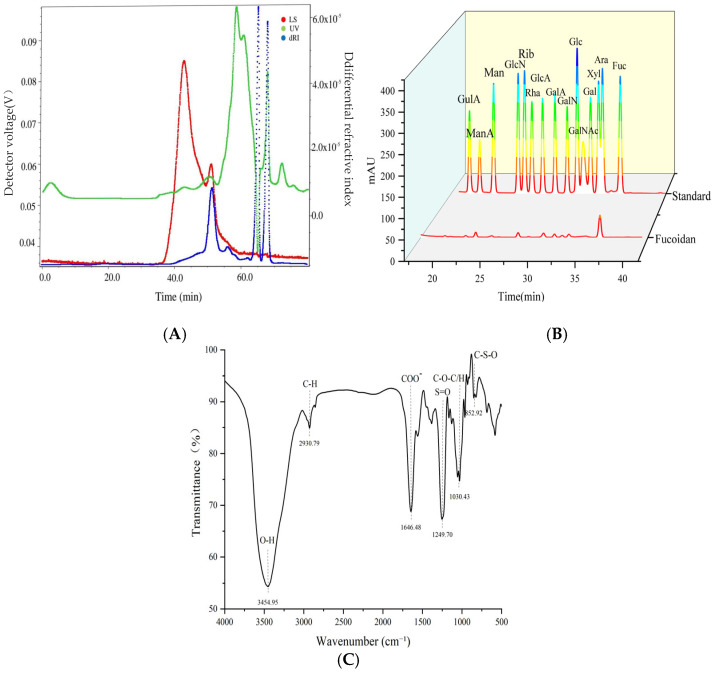
Structural characterization of kelp fucoidan. Note: (**A**) molecular weight of kelp fucoidan; (**B**) monosaccharide composition of kelp fucoidan; (**C**) FTIR of kelp fucoidan.

The structural characterization of kelp fucoidan is helpful to clarify its mechanism of action in repairing the immunosuppression caused by cyclophosphamide and restoring the intestinal microecological balance by regulating the intestinal flora and immune pathways, providing a scientific basis for its immunomodulatory treatment [34]. The molecular weight of fucoidan was determined by HPGPC-MALLS technology. Its characteristics are shown in Figure 1A. The number-average molecular mass (Mn) was 246 KDa, the peak molecular mass (Mn) was 299.31 KDa, and the heavy average molecular mass (Mw) was 555.09 KDa. The Mz was 1140.57 KDa, and the Mw/Mn was 2.25 with a polydispersion coefficient. The rotation radius (Rz) was 53.50 nm. Under normal circumstances, Mw is commonly used as the characterization result of molecular weight in industry. Nguyen, T. et al. [35] extracted kelp fucoidan by enzymatic hydrolysis, resulting in a molecular weight range of 300–800 KDa, which is higher than that of the kelp fucoidan in this study. This is mainly due to the differences in algal species sources, growth environments, and extraction processes (such as temperature and acid–base treatment).

As shown in Figure 1B, kelp fucoidan mainly consists of fucose (42.55%), rhamnose (2.67%), galactose (39.14%), xylose (1.84%), mannose (3.85%), and glucuronic acid (9.95%). Among these, fucose is the main monosaccharide, and fucoidan does not contain glucose. It is speculated that glucose was eluted first in the purification process. This result is consistent with the findings of Nguyen, T.T et al. [35], who reported that the monosaccharide composition of kelp fucoidan (*latissima*) is a mixture of fucose and galactomannan.

The FTIR spectrum of fucoidan in kelp is shown in Figure 1C. The signal has a wide and strong peak near 3400 cm^−1^, which is due to the stretching of O-H. This phenomenon is particularly obvious in the spectrum of polysaccharides. The band near 2930 cm^−1^ represents the stretching vibration of C-H, indicating the presence of a methyl absorption peak. The asymmetric stretching vibrations corresponding to C=O and C-O-C at 1640 cm^−1^ and 1070 cm^−1^, respectively, indicate the presence of aldehyde acid. The absorption peak near 1640 cm^−1^ may originate from the water molecules adsorbed by polysaccharides during the extraction or drying process. The absorption at 1250 cm^−1^ and 854 cm^−1^ indicates the presence of sulfates in the components, which suggest the sulfate pattern of fucoglycan, representing the presence of 4-O-sulfated Fuc or GalNAc. These characteristic functional groups are highly consistent with the previous results of physical and chemical property analysis [36].

### 3.2. Effects of Each Dose Group of Fucoidan on the Immune Indicators of Mice

#### 3.2.1. Analysis of DAI Index and Body Weight in Immunosuppressed Mice

**Figure 2:**
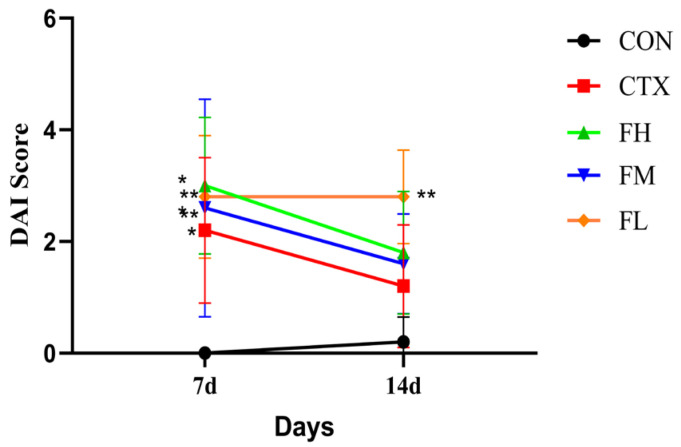
Changes in DAI index in mice. Note: * *p* < 0.05, ** *p* < 0.01: Compared with the CON group.

In order to explore the effects of fucoidan on immunosuppressed mice, we first established a immunosuppressed mice model induced by CTX. Subsequently, fucoidan was administered orally by gavage. CTX treatment led to weight loss, soft feces, and bloody stools in mice. Supplementation with fucoidan significantly improved fecal conditions. Figure 2 displays the DAI scores; after seven days, DAI indices for the CTX, FH, FM, and FL groups increased significantly compared with the CON group (*p* < 0.01), indicating successful modeling. However, there were no prominent differences between the FH, FM, and FL groups and the CTX group (*p* > 0.05).

Body weight can reflect the immune ability of mice to some extent. Linbin, S. et al. [37] studied that on the 7th day of administration, the white blood cell count in the CTX group (1.74 ± 0.49) and the administration group (2.40 ± 0.329) decreased significantly compared to the blank group (5.57 ± 1.10), while the administration group increased significantly compared to the CTX group. Decreased white blood cells directly affect immune cell production, leading to immune suppression and further weight loss accompanied by metabolic abnormalities. As shown in Table 3, there was no prominent difference in the body weights of the five groups of mice in the beginning of the experiment. After 7 days, the body weight of the mice in the CON group increased and was heavier than that of the mice in the other experimental groups, which indicates that the experimental model was successful and verifies that CTX causes the body weight of the mice to decrease. After continuous administration for 14 days to the experimental groups, the changes in the body weight of the mice in the FH and FM groups varied, with a higher increase in the FH group. Nevertheless, there was no disparity between the experimental group and the model group. This might imply that the acute toxicity of CTX was most significant at 7 days (inhibiting weight gain outside the CON group), but after 14 days, the model group partially compensated and recovered, narrowing the difference with the experimental group. Moreover, the intervention effects of the FH or FM groups may have been masked by the natural repair after CTX clearance, or may have only accelerated the recovery process (without exceeding the self-healing upper limit of the model group).

#### 3.2.2. Analysis of Immune Organs in Immunosuppressed Mice

It is critical for animals to have thymus and spleen organs, which are commonly used as key factors to evaluate their immune function. Yi-ming, X. et al. [38] found that high blood sugar and insulin deficiency led to thymic atrophy, with a significant reduction in the cortical/medullary area ratio (1.8 ± 0.89), which was significantly different from that of the control group (6.38 ± 1.97) (*p* < 0.01). The number of cortical thymocytes significantly decreased, and the cortex became sparse. The density of thymocytes per unit area in the cortex (43.14 ± 1.34) was significantly reduced, and there was a significant difference compared to the control group (59.95 ±0.86) (*p* < 0.01). Along with a decrease in T cells, high blood sugar also leads to splenic lesions (such as white pulp atrophy and follicle reduction). This confirms that the status of thymus and spleen is closely related to systemic immunity. In Table 4, CTX intervention led to a prominent increase in the pancreatic index (*p* < 0.01) and a noticeable decrease in the thymus index (*p* < 0.05) compared to the CON group, which implies the experimental model was established successfully. Yet, there were no remarkable differences found between the experimental groups and the model group. To some extent, this may have been due to insufficient administration time, which made it difficult to visually observe changes in the index.

#### 3.2.3. Analysis of Cytokine Levels in the Serum of Immunosuppressed Mice

**Figure 3:**
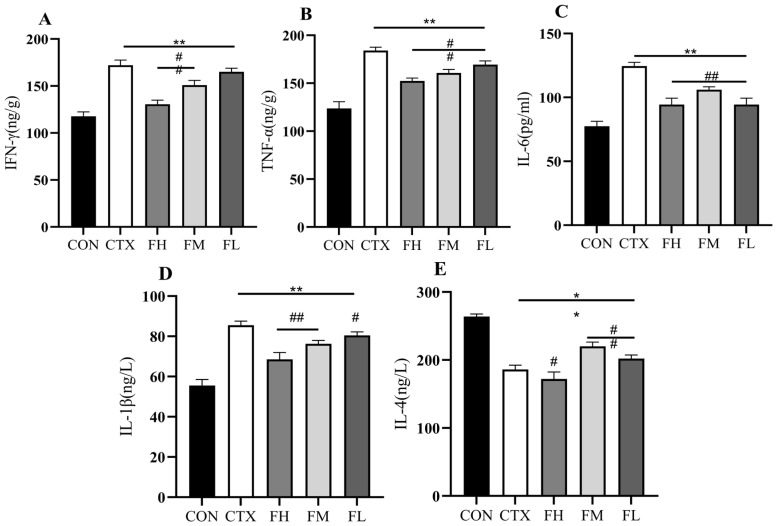
Immune indexes in serum of mice. (**A**) Level of IFN-γ, (**B**) level of TNF-α, (**C**) level of IL-6, (**D**) level of IL-1β, and (**E**) level of IL-4. Note: * *p* < 0.05, ** *p* < 0.01: compared with the CON group; # *p* < 0.05, ## *p* < 0.01: compared with the CTX group.

Cytokines are proteins and glycoproteins with active functions, such as IFN-γ, TNF-α, IL-1β, and IL-6, that play a vital role in immune responses [39]. Fucoidan can regulate macrophage polarization (M1/M2), dendritic cell maturation, and NK cell activity by activating the TLR/NF-κB and MAPK signaling pathways, thereby balancing pro-inflammatory (TNF-α, IL-6) and anti-inflammatory (IL-10) cytokines. Different fucoidan doses on immunocompromised mice have different effects, which are shown in Figure 3A–E. In the CON group, the concentrations of IFN-γ, TNF-α, IL-6, IL-1β, and IL-4 in serum were 671.66 ng/L, 680.91 ng/L, 77.46 ng/L, 55.54 ng/L, and 263.75 ng/L, respectively. After CTX intervention, the concentrations of IFN-γ, TNF-α, IL-6, and IL-1β significantly increased to 979.39 ng/L, 1008 ng/L, 124.55 ng/L, and 85.51 ng/L (*p* < 0.01), while the concentration of IL-4 significantly decreased to 186.11 ng/L (*p* < 0.01). In the experimental groups, the serum concentrations of IFN-γ, TNF-α, IL-6, IL-1β, and IL-4 were as follows: FH group— 814.31 ng/L, 840.90 ng/L, 94.46 ng/L, 68.55 ng/L, and 172.24 ng/L; FM group—878.01 ng/L, 912.10 ng/L, 106.08 ng/L, 76.33 ng/L, and 220.10 ng/L; and FL group—756.44 ng/L, 944.57 ng/L, 94.46 ng/L, 80.44 ng/L, and 202.15 ng/L. The data indicate that serum levels of IFN-γ, TNF-α, IL-6, and IL-1β decreased, while IL-4 levels are on the rise in the FH, FM, and FL groups. Akao et al. [40] found that fucose-modified polymers could counteract severe hepatitis induced by lipopolysaccharide (LPS). Fucose is one of the main components of the above-mentioned polysaccharides. However, fucose administration significantly reduced the levels of TNF-α and IL-1β (*p* < 0.05). This study (CTX model) shows that fucoidan promotes immune repair by reducing Th1 cytokines (IFN-γ/TNF-α) and increasing Th2-type IL-4, and may restore the immune function damaged by CTX by regulating the Th2/Treg balance. In the Akao study (LPS model), fucose-modified polymers mainly exert anti-inflammatory effects by inhibiting TNF-α/IL-1β, directly targeting the TLR4-NF-κB pathway to suppress LPS-driven inflammatory storms. Although the two have different directions of immune regulation and mechanisms of action, both have demonstrated that specific polysaccharide components can regulate immune responses, significantly reduce the levels of pro-inflammatory cytokines (TNF-α, IL-1β, etc.), and have repair or protective effects on immune system damage, showing the potential therapeutic value of polysaccharides in immune regulation.

#### 3.2.4. Effect of Serum Immunoglobulin Content in Immunosuppressed Mice

**Figure 4:**
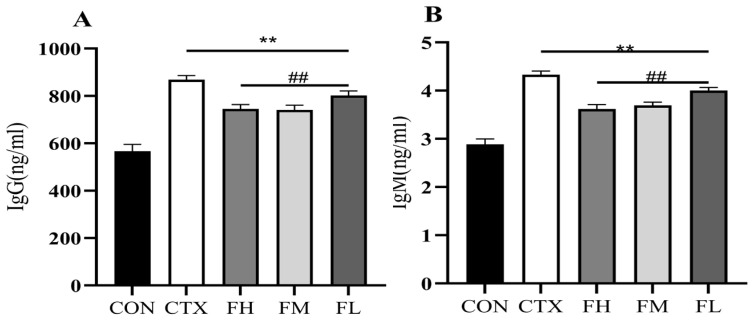
Serum IgG and IgM levels of mice. (**A**) Level of IgG, (**B**) level of IgM. Note ** *p* < 0.01: compared with the CON group; ## *p* < 0.01: compared with the CTX group.

The body plays a critical role in immune responses to antibodies produced from viruses, bacteria, and toxins. Among these, IgG and IgM are momentous components to resistance against external infections and the enhancement of immune function [41]. The levels of IgG and IgM are regarded as direct indicators of the body’s immune ability [42]. As shown in Figure 4, the serum standards of IgG and IgM in the CON group were 527.27 ng/mL and 2.88 ng/mL individually. After CTX interference, the IgG and IgM levels changed a lot, increasing to 869.12 ng/mL and 4.33 ng/mL (*p* < 0.01). In contrast, the FH group recorded IgG and IgM levels of 745.24 ng/mL and 3.62 ng/mL individually, while the FM group showed levels of 741.34 ng/mL and 3.69 ng/mL. The FL group exhibited slightly higher values, with IgG at 801.98 ng/mL and IgM at 4.00 ng/mL. These results indicate that fucoidan significantly reduced IgG and IgM levels in the FH, FM, and FL groups compared to the CTX group (*p* < 0.01). The research by Huang et al. [43,44,45,46,47] indicates that β-glucan and others can activate B cells, enhance IgG (especially IgG1/IgG2a), and short-term IgM. In this study, CTX, as an immunosuppressant, directly killed rapidly proliferating B cells during the acute phase, resulting in a decrease in IgG/IgM levels. However, during the recovery phase (7–14 days later), it triggered a compensatory immune reconstitution response, manifested as feedback proliferation of B cells and Th2-type immune shift (elevated IL-4), thereby causing a rebound in antibody levels. Fucoidan intervention effectively alleviates the compensatory antibody increase induced by CTX by activating the secretion of anti-inflammatory factors by regulatory B cells (Breg) and T cells (Treg), regulates the intestinal flora to promote the production of short-chain fatty acids, and inhibits the excessive differentiation of plasma cells through the GPR43 receptor, thereby restoring the immune state to the physiological level. This reflects its “immune normalization” characteristic.

### 3.3. Effects of Each Dose Group of Fucoidan on the Intestinal Flora of Immunocompromised Mice

#### 3.3.1. Analysis of Intestinal Flora Richness

**Figure 5:**
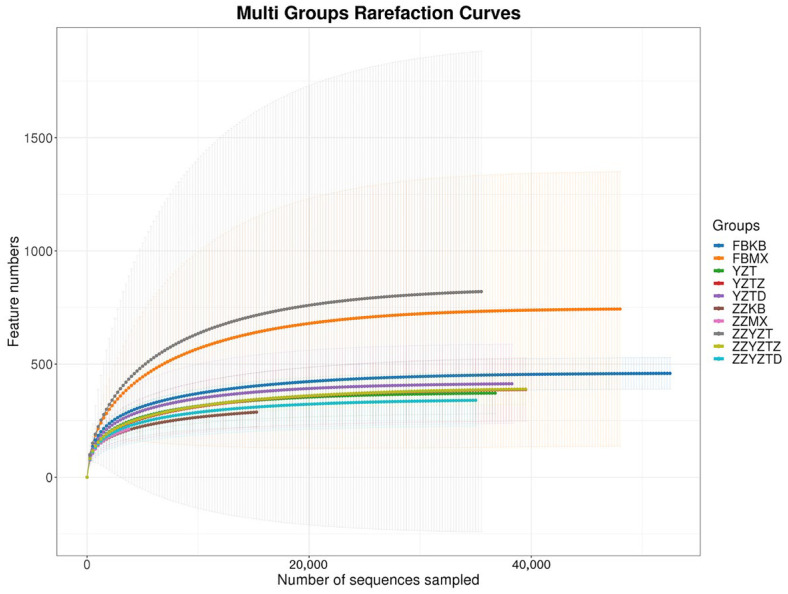
Dilutive curve based on OTU levels of mice intestinal flora. In feces: FBKB: CON group, FBMX: CTX group, YZT: FH group, YZTZ: FM group, YZTD: FL group; among colonic contents: ZZKB: CON group, ZZMX: CTX group, ZZYZT: FH group, ZZYZTM: FM group, ZZYZTD: FL group.

**Figure 6:**
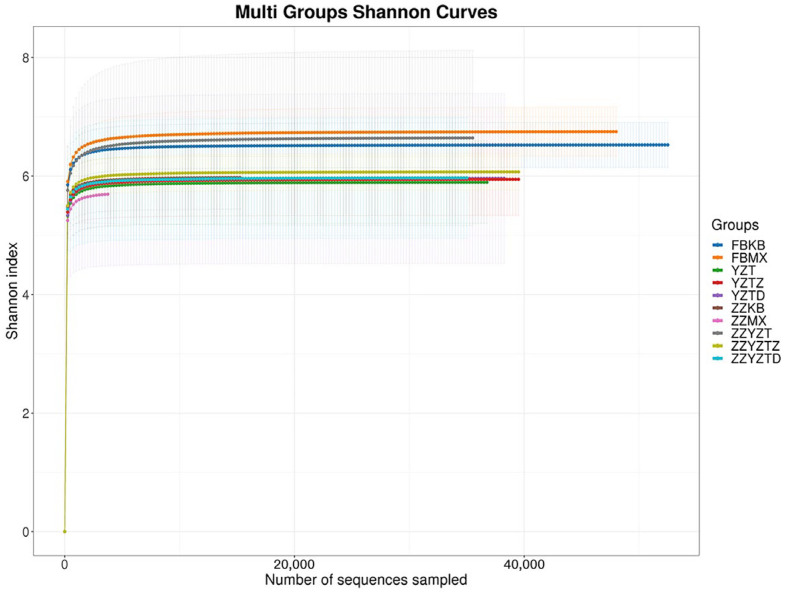
Shannon index based on OTU levels of mice intestinal flora. In feces: FBKB: CON group, FBMX: CTX group, YZT: FH group, YZTZ: FM group, YZTD: FL group; among colonic contents: ZZKB: CON group, ZZMX: CTX group, ZZYZT: FH group, ZZYZTM: FM group, ZZYZTD: FL group.

The richness of species is often reflected in the dilutive curve and Shannon index, which are not only used to estimate the number of species represented by sequencing data but also to evaluate whether the data is sufficient to capture the species diversity of the sample [48,49]. As shown in Figure 5 and Figure 6, similar to the research results of Juan Huang et al. [50], with the increase in sequencing depth, the rarefaction curve and Shannon index at the OTU level of the mouse intestinal microbiota gradually plateaued. This indicates that the sequencing depth is adequate, as the number of characteristic species no longer increases with further sequencing, demonstrating that the data are sufficient to cover the majority of microbial species and provide reliable representativeness.

#### 3.3.2. Analysis of the Diversity of Intestinal Flora in Mice

**Figure 7:**
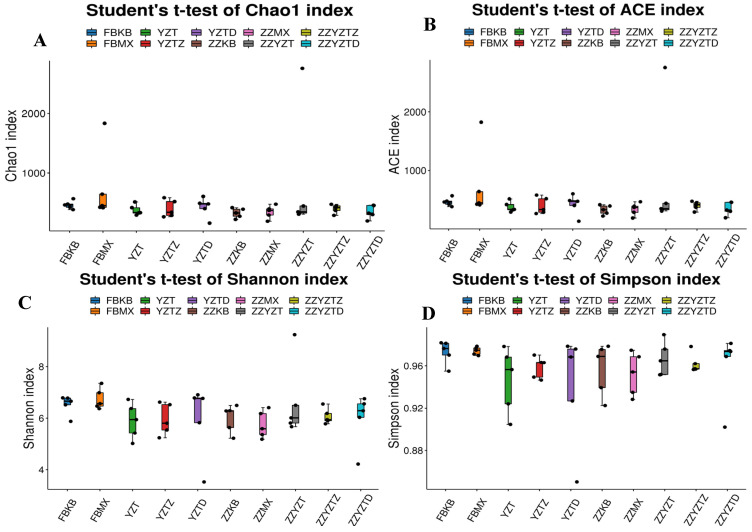
Difference analysis of alpha diversity index in intestinal flora of mice. (**A**) Chao1 index, (**B**) ACE index, (**C**) Shannon index, and (**D**) Simpson index. In feces: FBKB: CON group, FBMX: CTX group, YZT: FH group, YZTZ: FM group, YZTD: FL group; among colonic contents: ZZKB: CON group, ZZMX: CTX group, ZZYZT: FH group, ZZYZTM: FM group, ZZYZTD: FL group.

Figure 7 demonstrates the results of the alpha diversity analysis of intestinal flora, revealing that the α-diversity indices (Chao1, Ace, Shannon, and Simpson) for each experimental group remained unchanged. This implies that the addition of fucoidan had no effect on the richness or community diversity of the intestinal flora in the colonic contents and feces of immunocompromised mice.

**Figure 8:**
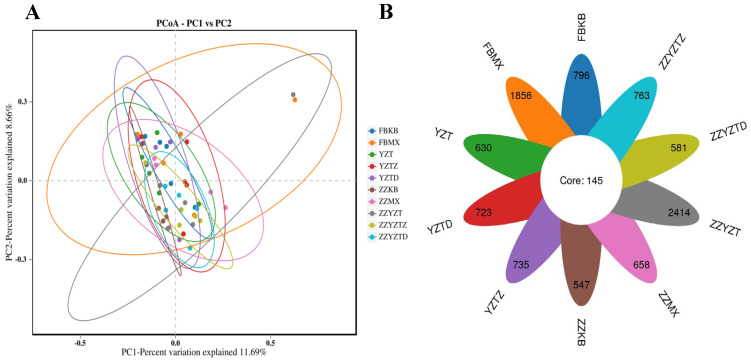
PCoA analysis and OTU quantity distribution of intestinal flora. (**A**) PCoA analysis, (**B**) OTU quantity distribution. In feces: FBKB: CON group, FBMX: CTX group, YZT: FH group, YZTZ: FM group, YZTD: FL group; among colonic contents: ZZKB: CON group, ZZMX: CTX group, ZZYZT: FH group, ZZYZTM: FM group, ZZYZTD: FL group.

Beta diversity analysis can be represented by the unweighted UniFrac principal coordinate (PCoA). The closer the points on the coordinate graph are, the higher the similarity between the samples [51]. There was no significant difference in species diversity among the treatment groups (Figure 8A). In total, 50 samples were sequenced in this experiment, yielding 3,378,396 sequences, with an average of 61,774 sequences per sample. A total of 9848 OTUs were identified, of which 145 were shared. The colonic contents showed a higher number of specific OTUs compared to feces. In fecal samples, the FBKB, FBMX, YZT, YZTD, and YZTZ groups contained 796, 1,856, 630, 723, and 735 specific OTUs respectively. In colonic contents, the ZZKB, ZZMX, ZZYZT, ZZYZTD, and ZZYZTZ groups contained 547, 658, 2414, 581, and 763 specific OTUs, respectively (Figure 8B). These results imply that CTX and fucoidan intervention had a measurable effect on the OTU composition of the intestinal microecology in immunocompromised mice. Huang et al. [50]. found that CTX treatment significantly altered the β diversity of the colonic contents and fecal microbiota in mice, while the microbiota structure approached that of the normal group after SA intervention, indicating that SA could effectively alleviate the intestinal microbiota disorder induced by CTX. This result is relatively consistent with this study. The β-diversity changes in colonic contents are more significant than those in feces, suggesting that fucoidan had a more direct regulatory effect on the local intestinal flora.

#### 3.3.3. Analysis Based on the Gate Classification Level

**Figure 9:**
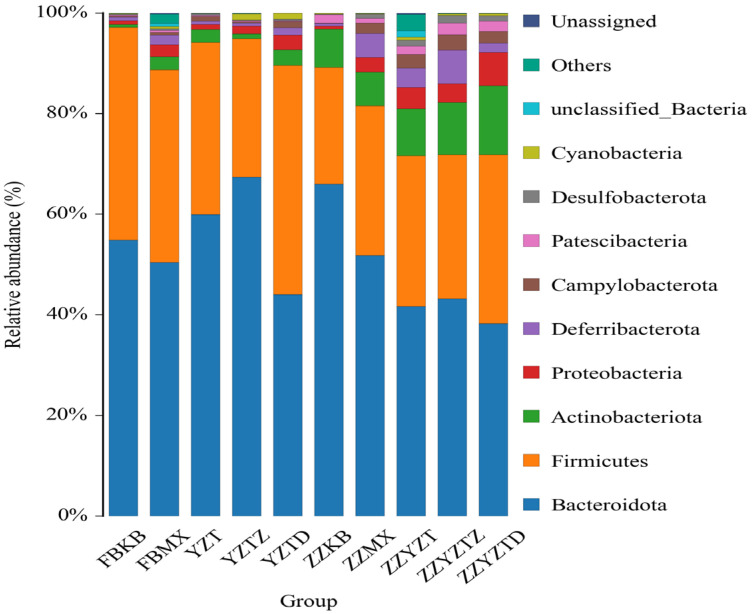
Difference analysis of flora abundance in each treatment group. In feces: FBKB: CON group, FBMX: CTX group, YZT: FH group, YZTZ: FM group, YZTD: FL group; among colonic contents: ZZKB: CON group, ZZMX: CTX group, ZZYZT: FH group, ZZYZTM: FM group, ZZYZTD: FL group.

As depicted in Figure 9, the fecal microbiota of normal mice primarily consisted of *Bacteroidota* (54.8%), *Firmicutes* (42.3%), and *Actinobacteriota* (0.57%). The abundances of *Bacteroidota* and *Firmicutes* in feces decreased to 50.37% and 38.28%, while *Actinobacteriota* increased to 2.67% because of the CTX intervention. In the fucoidan treatment groups YZT, YZTZ, and YZTD, there was an increase in *Bacteroidota* and *Actinobacteriota* in feces, and a reduction of *Firmicutes* in the YZTZ group back to normal levels, with the dose-dependent group showing the most pronounced effects. In the colonic contents of normal mice, the microbiota mainly contained *Bacteroidetes* (65.94%), *Firmicelles* (23.22%), and *Actinomycetes* (7.61%). After CTX intervention, the *Bacteroidota* decreased to 51.8%, whereas *Firmicutes* and *Actinobacteriota* increased to 29.69% and 7.97%, respectively. Fucoidan treatment resulted in reduced *Bacteroidota* in the ZZYZT, ZZYZTM, and ZZYZTD groups but increased *Actinobacteriota*, with a decrease in *Firmicutes* noted in the ZZYZTM group. Research by Xie et al. [52] demonstrated that antibiotics significantly decreased the proportions of *Firmicutes* and *Bacteroidota* in mouse intestines, while fucoidan intervention notably increased *Bacteroidota* levels, aligning with our study’s results. Reports suggest that *Bacteroidota* can mitigate intestinal inflammation and strengthen the intestinal barrier. GAO et al. [53] observed that fucoidan enhances *Firmicutes* and *Bacteroidota* ratios, alleviating mouse intestinal inflammation. Liang et al. [54] demonstrated that the thick-walled bacteria phylum has both anti-inflammatory and immunosuppressive effects simultaneously. Studies have found that higher abundances of *Bacteroides* mean more significant the side effects on the learning and cognitive abilities of the body, and *Bacteroides* is positively correlated with inflammatory responses [55]. Thus, fucoidan may modify colonic content microbiota and improve intestinal barrier functionality.

#### 3.3.4. Analysis of Differences in Intestinal Flora

**Figure 10:**
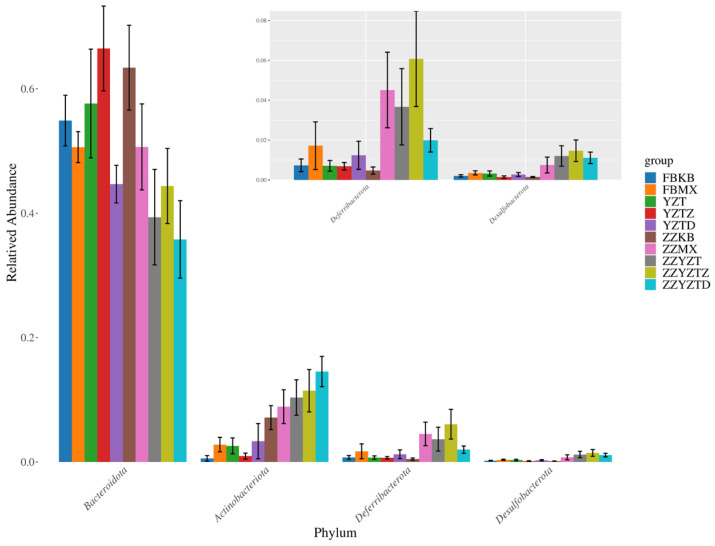
Differences analysis of intestinal flora in different phyla of each treatment group. In feces: FBKB: CON group, FBMX: CTX group, YZT: FH group, YZTZ: FM group, YZTD: FL group; among colonic contents: ZZKB: CON group, ZZMX: CTX group, ZZYZT: FH group, ZZYZTM: FM group, ZZYZTD: FL group.

As shown in Figure 10, *Bacteroidota* exhibited the highest abundance across all groups. The abundance of *Bacteroidota* increased in the colonic contents but decreased in feces because of CTX intervention. In contrast, the abundances of *Actinobacteriota*, *Deferribacterota*, and *Desulfobacterota* were higher in colonic content than in feces. CTX intervention caused significant changes in these microbial populations, which were mitigated by fucoidan treatment. The abundance patterns indicated that *Actinobacteriota*, *Deferribacterota*, and *Desulfobacterota* were excreted in small amounts via feces, while *Bacteroidota* was predominantly eliminated through fecal excretion. CTX intervention resulted in increased abundances of *Actinobacteriota*, *Deferribacterota*, and *Desulfobacterota*, along with a decrease in *Bacteroidota*. However, fucoidan treatment effectively reversed these changes, contributing to the restoration of intestinal microbial stability in mice.

#### 3.3.5. Analysis Based on the Classification Level of Families and Genera

**Figure 11:**
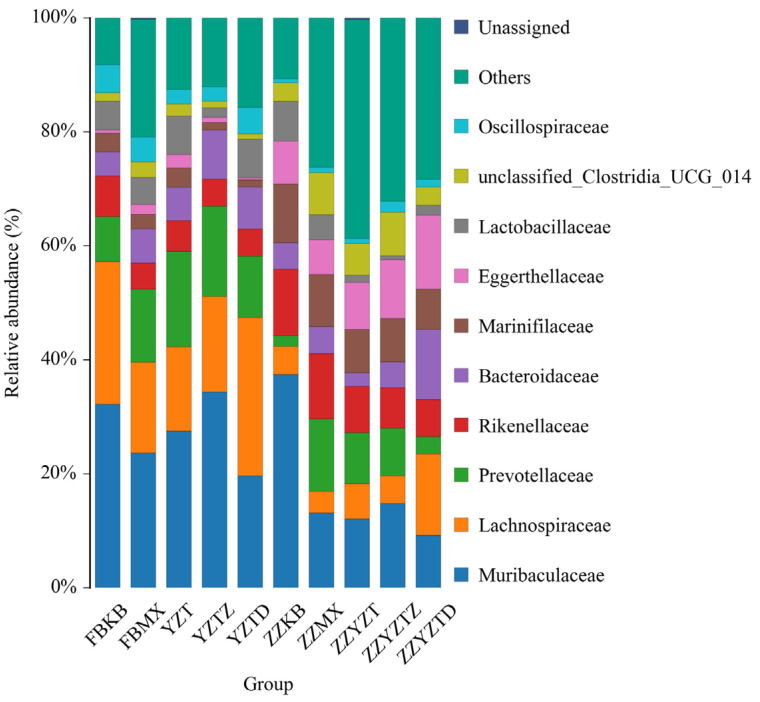
Abundance analysis of bacteria from different families. In feces: FBKB: CON group, FBMX: CTX group, YZT: FH group, YZTZ: FM group, YZTD: FL group; among colonic contents: ZZKB: CON group, ZZMX: CTX group, ZZYZT: FH group, ZZYZTM: FM group, ZZYZTD: FL group.

As shown in Figure 11, the intestinal flora in the feces of normal mice was primarily made up of *Muribaculaceae* (32.18%), *Lachnospiraceae* (25.04%), *Prevotellaceae* (7.93%), *Rikenellaceae* (7.11%), *Bacteroidaceae* (4.18%), *Marinifilaceae* (3.38%), *Eggerthellaceae* (0.55%), and *Lactobacillaceae* (5.02%). Among these, *Muribaculaceae*, *Prevotellaceae*, *Rikenellaceae*, and *Bacteroidaceae* belong to the phylum *Bacteroidota*; Phylum *Firmicutes* includes *Lachnospiraceae* and *Lactobacillaceae*; *Marinifilaceae* and *Eggerthellaceae* belong to the phylum *Actinobacteriota* [56,57,58,59,60]. Due to the CTX, the abundances of *Muribaculaceae*, *Lachnospiraceae*, *Rikenellaceae*, *Marinifilaceae*, and *Lactobacillaceae* separately decreased to 23.65%, 15.95%, 4.58%, 2.56%, and 4.73%, while the abundances of *Prevotellaceae*, *Bacteroidaceae*, and *Eggerthellaceae* individually increased to 12.79%, 5.99%, and 1.72%. Fucoidan treatment improved the abundance of these bacterial communities to varying degrees, with the high-dose group showing the most significant effects. In the colonic contents of normal mice, the intestinal flora was predominantly composed of *Muribaculaceae* (37.42%), *Lachnospiraceae* (4.89%), *Prevotellaceae* (1.93%), *Rikenellaceae* (11.64%), *Bacteroidaceae* (4.66%), *Marinifilaceae* (10.24%), *Eggerthellaceae* (7.60%), and *Lactobacillaceae* (6.99%). In the CTX group, the abundances of *Muribaculaceae*, *Lachnospiraceae*, *Rikenellaceae*, *Marinifilaceae*, *Lactobacillaceae*, and *Eggerthellaceae* declined to 13.10%, 3.83%, 11.47%, 9.14%, 4.44%, and 6.08%, whereas the abundances of *Prevotellaceae* and *Bacteroidaceae* increased to 12.72% and 4.71%, respectively. Fucoidan treatment also restored the abundance of these bacterial communities to varying degrees.

**Figure 12:**
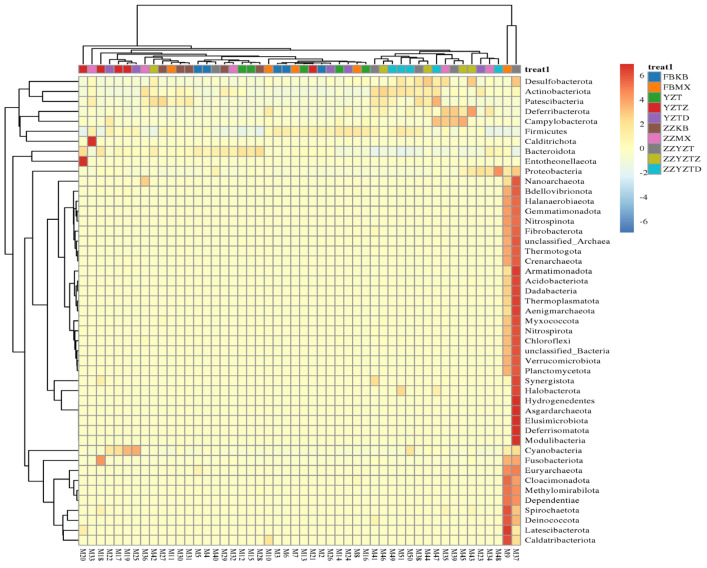
Cluster analysis of horizontal intestinal flora. In feces: FBKB: CON group, FBMX: CTX group, YZT: FH group, YZTZ: FM group, YZTD: FL group; among colonic contents: ZZKB: CON group, ZZMX: CTX group, ZZYZT: FH group, ZZYZTM: FM group, ZZYZTD: FL group.

As shown in Figure 12, based on the clustering heat map of the intestinal microbiota at the genus level, it was found that CTX treatment had the most significant effect on the intestinal microbiota. However, fucoidan treatment in the FH, FM, and FL groups significantly alleviated CTX-induced dysbiosis in the intestinal flora of mice.

### 3.4. Determination of SCFAs in Feces and Colonic Contents of Immunocompromised Mice

#### 3.4.1. Effects of Each Dose Group of Fucoidan on SCFAs in the Colonic Contents of Mice

In terms of the microbiome, fucoidan sulfate groups directly inhibit pathogen adhesion and biofilm formation, while acting as prebiotics to promote the proliferation of beneficial bacteria (such as lactic acid bacteria) and generate SCFAs, which are then absorbed by the small intestine and subsequently enter the colon to serve as an energy source for cells [61,62]. SCFAs have the functions of reducing the pH value of the colon, inhibiting pathogenic bacteria, and regulating the function of the intestinal mucosal barrier [63]. Studies have shown that propionic acid can reduce the surge of inflammatory factors associated with alcoholic liver disease by lowering cholesterol levels [64]. Additionally, Jiang et al. [65] found that purple daisy polysaccharides can promote the production of n-butyric acid by alleviating alcohol-induced intestinal dysbiosis, thereby maintaining the integrity of the intestinal barrier. SCFAs are synthesized by specific bacterial communities: propionic acid is primarily produced by *Actinobacteriota*, *Bacteroidota*, and *Firmicutes*; acetic acid is predominantly formed by *Bacteroidota*; butyric acid is synthesized by both *Bacteroidota* and *Firmicutes*; and valeric acid is mainly produced by *Bacteroidota* [63]. In order to determine the effects of various doses of fucoidan on the intestinal microbiota metabolism of mice, it is necessary to measure the contents of SCFAs in the colonic contents.

**Figure 13:**
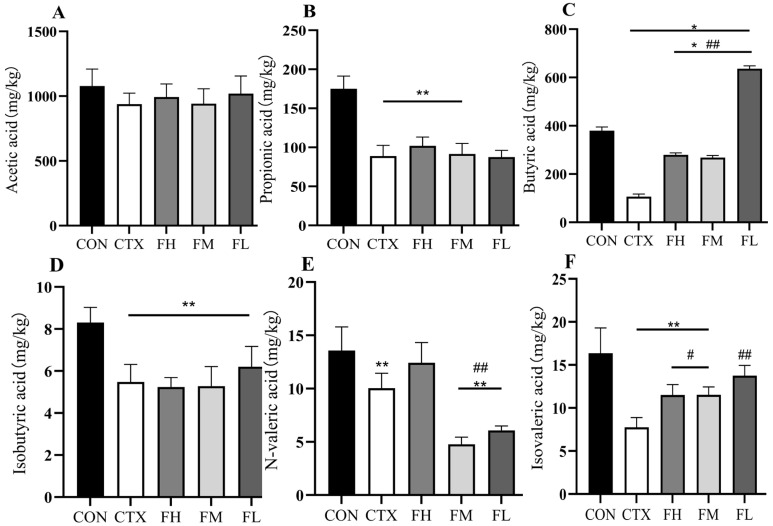
Analysis of SCFAs in colon contents of mice. (**A**) Content of acetic acid, (**B**) content of propionic acid, (**C**) content of butyric acid, (**D**) content of isobutyric acid, (**E**) content of valeric acid, and (**F**) content of isovaleric acid. Note: * *p* < 0.05, ** *p* < 0.01: compared with the CON group; # *p* < 0.05, ## *p* < 0.01: compared with the CTX group.

The results (Figure 13) show that the levels of propionic acid, n-butyric acid, isobutyric acid, n-valeric acid, and isovaleric acid in the colon of the CTX group were significantly lower than those of the CON group (*p* < 0.01). However, the intervention of fucoidan significantly restored the levels of n-butyric acid, n-valeric acid, and isovaleric acid (Figure 13C,E,F). Specifically, the FL group induced a significant increase in n-butyric acid (*p* < 0.01, Figure 13C), while the FH group was the most effective at increasing n-valeric acid levels (*p* < 0.01, Figure 13E). Thus, CTX intervention led to a marked depletion of SCFAs, whereas FH, FM, and FL interventions reversed this decline. Among the interventions, FL best increased the level of n-butyric acid, while FH maximized the level of n-valeric acid. These SCFAs changes align with the alterations in bacterial composition. There was a significant reduction in the abundance of *Bacteroidota* and *Firmicutes*, which resulted in decreased levels of butyric acid, isobutyric acid, valeric acid, and isovaleric acid because of the CTX intervention. Similarly, Liu et al. [66] discovered that DSS treatment significantly reduced the levels of butyric acid, and valeric acid increased with fucoidan in the cecum. Additionally, the DSS group exhibited a reduction in propionic acid and isovaleric acid levels, while the FUC group significantly elevated acetic acid, propionic acid and isovaleric acid levels (*p* < 0.01). In conclusion, FH, FM, and FL can be metabolized by intestinal microbiota, modulating SCFA concentrations and ultimately improving intestinal health.

#### 3.4.2. Effects of Each Dose Group of Fucoidan on SCFAs in Mouse Feces

**Figure 14:**
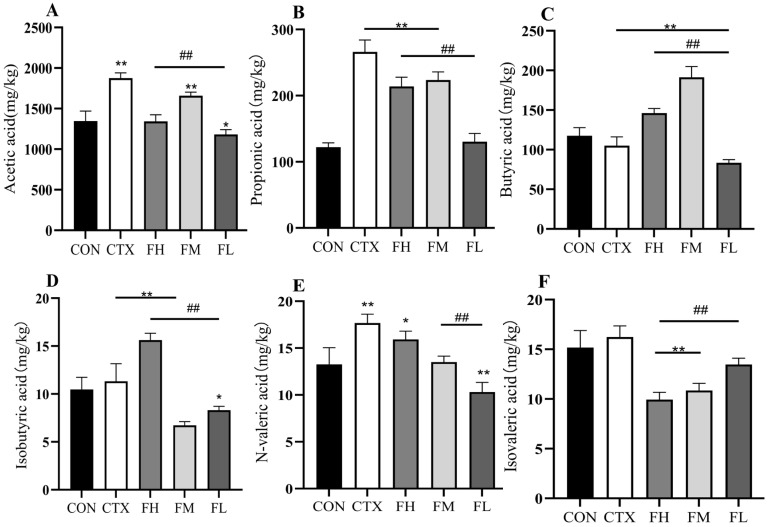
Analysis of SCFAs in feces of mice. (**A**) Content of acetic acid, (**B**) content of propionic acid, (**C**) content of butyric acid, (**D**) content of isobutyric acid, (**E**) content of valeric acid, and (**F**) content of isovaleric acid. Note: * *p* < 0.05, ** *p* < 0.01: compared with the CON group ## *p* < 0.01: compared with the CTX group.

According to Figure 14, compared with the CON group, the CTX group displayed a significant increase in the fecal contents of acetic acid, propionic acid, n-valeric acid, and isobutyric acid (*p* < 0.01), while the content of n-butyric acid was significantly reduced (*p* < 0.01). In contrast, the content of isovaleric acid showed no significant change (*p* > 0.05). Intervention with FH, FM, and FL effectively reversed these alterations. Specifically, the FM group demonstrated the most pronounced effect in increasing acetic acid and n-butyric acid levels while reducing propionic acid and isobutyric acid levels. The FH group had the greatest effect on increasing n-valeric acid, whereas the FL group was most effective in elevating isovaleric acid levels.

Notably, following CTX intervention, the fecal SCFAs profile (excluding n-butyric acid) exhibited an opposite trend compared to that in colonic contents. This may be attributed to the excretion of intestinal metabolites through feces. It can be inferred from this that the reduction in excretion led to the content of n-butyric acid in feces being lower than that in colonic contents. These findings suggest that CTX intervention disrupts the balance of colonic and fecal metabolites, while FH, FM, and FL interventions can effectively restore this equilibrium.

## 4. Conclusions

This research revealed that kelp fucoidan exhibited dual microbiota restoration and immunomodulatory effects in CTX-induced immunosuppressed mice. Through immunological assays and 16S rRNA sequencing, three main mechanisms of actions were identified. Kelp fucoidan (1) significantly (*p* < 0.05) enhanced the abundance of probiotics (*Bifidobacterium* and *Lactobacillus*) but restrained opportunistic pathogens (*E. coli* and *Enterococcus*); (2) supplemented immunostimulatory SCFA production; (3) restored splenic and thymic indices, along with enriching serum immunoglobulins (IgG/IgM) and IL-4. The measured 2.1-fold increase in *Bacteroidota*, combined with *firmicutes* decline, indicates kelp fucoidan potential in assisting mucin-degrading microflora that restore intestinal barrier functionality.

The findings of the present research reveal that kelp fucoidan is a promising therapeutic agent with exclusive benefits over conventional probiotic therapy. However, this study has certain limitations. The cyclophosphamide-induced immunosuppression model cannot fully simulate the complex immune deficiencies in humans, and the differences between stages were not considered. The causal relationship between gut microbiota and immune regulation has not been sufficiently validated through fecal microbiota transplantation or germ-free mouse experiments. Additionally, the oral bioavailability, long-term safety, and clinical individual variability of fucoidan have not been evaluated, which limits its clinical translation potential. Further research should focus on kelp fucoidan optimized dosage and effects in clinical trials, especially its therapeutic capacity to alleviate gut microbiota in patients.

## Figures and Tables

**Table 1:** Administration of different groups of mice.

Experimental Group	Number of Animals	Gavage Sample	Daily Gavage Dose	Duration of Administration
Blank control (CON)	5	-	Natural diet	14 days
Model group (CTX)	5	Normal saline	0.1 mL/10 g	14 days
Low-dose fucoidan (FL)	5	Fucoidan	50 mg/(kg·bw)	14 days
Medium-dose fucoidan (FM)	5	Fucoidan	100 mg/(kg·bw)	14 days
High-dose fucoidan (FH)	5	Fucoidan	150 mg/(kg·bw)	14 days

**Table 2:** DAI index evaluation.

Grade	Weight Loss (%)	Fecal Traits	Bloody Stool Condition
0	Lossless	Regular	No occult blood
1	1–5	Feces are soft but shaped	Positive occult blood
2	5–10	Very soft	Blood in the stool
3	10–15	Diarrhea	Hemafecia
4	>15	-	-

**Table 3:** Periodic changes in mice weight.

	CON	CTX	FH	FM	FL
0 d	17.72 ± 0.70	17.98 ± 0.30	18.44 ± 0.92	18.2 ± 1.07	18.48 ± 1.02
7 d	19.9 ± 1.18	17.32 ± 0.19 *	17.16 ± 0.99 **	17.22 ± 1.36 **	17.46 ± 1.21 *
14 d	20.36 ± 1.19	17.62 ± 0.84 **	17.56 ± 2.20 **	17.22 ± 2.06 **	16.34 ± 1.54 **

Note: * Indicates a significant difference compared with the blank group (*p* < 0.05), and ** indicates an extremely significant difference compared with the blank group (*p* < 0.01).

**Table 4:** Weights of spleen and thymus.

	CON	CTX	FH	FM	FL
Pancreatic organ coefficient	0.47 ± 0.06	1.25 ± 0.18 **	1.01 ± 0.28 **	1.02 ± 0.28 **	1.00 ± 0.25 **
Thymus organ co- efficient	0.19 ± 0.01	0.11 ± 0.04 *	0.10 ± 0.05 *	0.11 ± 0.06 *	0.09 ± 0.03 *

Note: * *p* < 0.05, ** *p* < 0.01: compared with the CON group.

## Data Availability

The original contributions presented in this study are included in the article. Further inquiries can be directed to the corresponding author.
